# Supplementary material for: Black Soybean Improves Vascular Function and Blood Pressure: A Randomized, Placebo Controlled, Crossover Trial in Humans
Source: Nutrients. 2020 Sep 10;12(9):2755. doi: 10.3390/nu12092755 (PMC7551904; doi:10.3390/nu12092755)
Supplement: Supplementary file 1 [file nutrients-12-02755-s001.pdf]

Table S1. Supporting data for anthropometric parameters of participants during the 4-week trial (Table 3).

**Test material**	**Placebo**			Black soybean		within-individual difference: BS-Placebo	
**Period**	**Start**	**End**		Start	End	Start	End
Body weight (kg)							
BS then placebo	63.9±4.0	64.5±4.1		63.8±4.1	63.6±4.1	-0.2	-0.9
Placebo then BS	63.7±4.0	64.0±4.0		64.2±3.9	64.2±3.8	0.5	0.2
Treatment effect	-	-		-	-	-0.2	-0.4
95% confidence interval						-7.5 to 8.4	-8.2 to 7.5
Welch's t test						*p*=0.9105	*p*=0.9250
BMI							
BS then placebo	22.7±1.1	22.9±1.1		22.6±1.1	22.6±1.1	-0.1	-0.3
Placebo then BS	23.4±1.4	23.5±1.4		23.6±1.3	23.6±1.3	0.2	0.1
Treatment effect	-	-		-	-	0.1	-0.1
95% confidence interval						-2.2 to 2.6	-2.5 to 2.3
Welch's t test						*p*=0.5523	*p*=0.4584
Body fat (%)							
BS then placebo	23.9±2.0	24.9±1.9		23.5±2.1	23.7±2.0	-0.4	-1.3
Placebo then BS	23.8±2.9	25.8±2.6		25.1±2.9	25.0±2.9	1.3	-0.8
Treatment effect	-	-		-	-	0.4	-1.0
95% confidence interval						-3.2 to 6.2	-5.8 to 3.7
Welch's t test						*p*=0.7348	*p*=0.3328
Visceral fat (%)							
BS then placebo	8.5±1.5	9.0±1.6		8.5±1.5	8.5±1.6	-0.1	-0.5
Placebo then BS	8.2±1.4	7.7±1.4	8.5±1.4	8.5±1.4	8.5±1.4	0.4	0.8
Treatment effect	-	-		-	-	0.1	0.2
95% confidence interval						-2.7 to 3.0	-2.8 to 3.1
Welch's t test						*p*=0.9234	*p*=0.5494
Biological age							
BS then placebo	36.0±3.3	37.3±3.4		35.5±3.1	35.5±3.4	-0.5	-1.7
Placebo then BS	36.5±4.8	37.4±4.7		38.7±4.8	38.5±4.8	2.2	1.2
Treatment effect	-	-		-	-	0.9	-0.3
95% confidence interval						-4.1 to 17.2	-8.4 to 7.9
Welch's t test						*p*=0.8886	*p*=0.4733
Basal metabolic rate (kcal/day)							
BS then placebo	1375±82	1371±81		1378±84	1370±81	3.6	-1.0
Placebo then BS	1361±73	1350±72		1355±71	1354±68	-6.5	4.7
Treatment effect	-	-		-	-	-1.5	1.9
95% confidence interval						-154.9 to 152.0	-147.9 to 151.6
Welch's t test						*p*=0.9801	*p*=0.9848

Estimated bone mass (kg)						
BS then placebo	2.6±0.2	2.6±0.2	2.6±0.1	2.6±0.2	0	0
Placebo then BS	2.6±0.1	2.7±0.1	2.6±0.1	2.6±0.1	0	-0.1
Treatment effect	-	-	-	-	0	0
95% confidence interval					-0.28 to 0.27	-0.30 to 0.24
Welch's t test					*p*=0.8404	*p*=0.947
Muscle mass (%)						
BS then placebo	46.0±3.2	45.8±3.1	46.2±3.2	45.9±3.1	0.1	0.1
Placebo then BS	45.5±2.8	44.4±2.7	45.1±2.7	45.2±2.7	-0.3	0.7
Treatment effect	-	-	-	-	-0.1	0.4
95% confidence interval					-6.0 to 5.8	-5.3 to 6.2
Welch's t test					*p*=0.8893	*p*=0.9766

BS; black soybean. Number of "BS then placebo" and "Placebo then BS" groups was 11 each and that of "Treatment effect" was 22.

Table S2. Supporting data for the effect of black soybean consumption on the vascular function (Table 4).

**Test material**	**Placebo**		Black soybean		within-individual difference: BS-Placebo	
**Period**	**Start**	**End**	Start	End	Start	End
Vascular function						
BS then placebo	52.0±3.1	52.3±2.9	54.1±3.8	50.9±3.4	2.1	-1.4
Placebo then BS	53.1±3.2	52.1±3.7	50.3±3.3	47.7±2.8	-2.8	-4.4
Treatment effect	-	-	-	-	-0.4	-2.9
95% confidence interval					-6.3 to 7.0	-3.6 to 9.3
Welch's t test					*p*=0.5439	*p*=0.0312
a wave						
BS then placebo	110.2±1.1	109.8±1.1	114.9±3.4	114.7±2.0	4.7	4.9
Placebo then BS	107.8±3.4	116.1±2.5	107.4±3.7	103.8±3.0	-0.5	-12.3
Treatment effect	-	-	-	-	2.1	-3.7
95% confidence interval					-8.5 to 4.2	-1.6 to 8.9
Welch's t test					*p*=0.5027	*p*=0.1656
b wave						
BS then placebo	−59.2±3.9	-54.1±5.1	−59.5±4.8	−62.9±3.4	-0.4	-8.8
Placebo then BS	−56.5±5.4	-63.9±5.1	−57.6±4.3	−61.7±2.6	-1.2	2.2
Treatment effect	-	-	-	-	-0.8	-3.3
95% confidence interval					-8.4 to 10.0	-5.3 to 12.0
Welch's t test					*p*=0.866	*p*=0.4406
c wave						
BS then placebo	−32.2±6.3	-30.5±7.9	−43.3±5.3	−31.7±5.3	-11.1	-1.3
Placebo then BS	−32.2±6.0	-42.2±7.1	−28.5±4.9	−22.8±4.2	3.6	19.4
Treatment effect	-	-	-	-	-3.7	9.0
95% confidence interval					-7.9 to 15.3	-21.9 to 3.8

Welch's t test					*p*=0.5196	*p*=0.1619
d wave						
BS then placebo	−49.5±6.8	-51.6±7.7	−66.6±6.3	−51.3±5.2	-17.2	0.4
Placebo then BS	−51.3±5.9	-58.6±8.9	−45.5±6.0	−37.0±3.9	5.7	21.6
Treatment effect	-	-	-	-	-5.7	11.0
95% confidence interval					-7.4 to 18.9	-24.7 to 2.7
Welch's t test					*p*=0.3863	*p*=0.056
Vascular waveform						
BS then placebo	3.8±0.3	3.9±0.5	4.4±0.5	3.5±0.4	0.5	-0.4
Placebo then BS	3.8±0.4	3.7±0.4	3.2±0.4	2.6±0.3	-0.6	-1.1
Treatment effect	-	-	-	-	0	-0.7
95% confidence interval					-0.8 to 0.9	-0.1 to 1.5
Welch's t test					*p*=0.9171	*p*=0.0392
Waveform score						
BS then placebo	44.2±2.9	43.1±5.3	37.7±4.2	47.5±3.5	-6.5	4.4
Placebo then BS	44.7±3.9	44.5±3.8	49.4±4.0	54.6±2.9	4.6	10.2
Treatment effect	-	-	-	-	-0.9	7.3
95% confidence interval					-15.3 to 0.7	-7.0 to 8.8
Welch's t test					*p*=0.9171	*p*=0.0368
Peripheral vascular health						
BS then placebo	64.0±3.6	61.6±6.0	56.2±2.9	65.1±3.2	-7.8	9.2
Placebo then BS	60.0±4.9	59.6±4.2	69.2±2.2	74.8±2.7	3.5	15.2
Treatment effect	-	-	-	-	0.7	9.3
95% confidence interval					-17.9 to -0.7	-8.3 to 6.9
Welch's t test					*p*=0.8569	*p*=0.017
Blood pressure						
Systolic blood pressure						
BS then placebo	119.9±5.4	127.5±4.4	128.0±5.0	116.5±4.4	8.1	-11.0
Placebo then BS	124.3±4.1	123.8±2.9	129.1±4.8	127.2±3.6	4.8	3.4
Treatment effect	-	-	-	-	6.4	-3.8
95% confidence interval					-13.0 to -0.3	-4.1 to 17.2
Welch's t test					*p*=0.2229	*p*=0.0392
Diastolic blood pressure						
BS then placebo	78.2±3.1	83.5±3.2	85.9±4.3	77.4±3.2	7.7	-6.1
Placebo then BS	84.2±2.9	81.6±3.6	83.2±3.7	84.5±2.2	-1.0	2.8
Treatment effect	-	-	-	-	3.4	-1.6
95% confidence interval					-3.7 to 10.7	-8.0 to 4.7
Welch's t test					*p*=0.6039	*p*=0.33
Central blood pressure						
BS then placebo	126.6±5.8	133.6±4.2	134.7±5.7	123.3±4.7	8.1	-10.4
Placebo then BS	132.2±4.2	131.6±5.2	133.4±6.8	132.0±4.3	1.2	0.4

Treatment effect	-	-	-	-	4.6	-5.0
95% confidence interval					-14.4 to 4.3	-6.7 to 16.0
Welch's t test					*p*=0.4133	*p*=0.2844

BS; black soybean. Number of "BS then placebo" and "Placebo then BS" groups was 11 each and that of "Treatment effect" was 22.

Table S3. Supporting data for the effect of black soybean consumption on the antioxidant activity (Table 5).

**Test material**	**Placebo**		Black soybean		within-individual difference: BS-Placebo	
**Period**	**Start**	**End**	Start	End	Start	End
Plasma						
NO_2/NO_3 (μM)						
BS then placebo	36.9±4.7	28.7±2.1	26.7±3.3	41.9±5.0	-10.2	13.1
Placebo then BS	21.4±4.9	30.4±1.9	31.4±4.4	28.9±2.4	10.1	-1.5
Treatment effect	-	-	-	-	-0.1	5.8
95% confidence interval					-9.1 to 9.8	-1.4 to 12.4
Welch's t test					*p*=0.9467	*p*=0.048
HEL (nM)						
BS then placebo	4.7±0.2	4.5±0.2	3.9±0.1	4.6±0.3	-0.8	0.2
Placebo then BS	3.9±0.2	4.7±0.2	4.7±0.3	4.3±0.2	0.8	-0.4
Treatment effect	-	-	-	-	0	-0.1
95% confidence interval					-0.4 to 0.5	-0.6 to 0.4
Welch's t test					*p*=0.858	*p*=0.6632
MPO (ng/mL)						
BS then placebo	88.3±4.5	95.7±6.2	88.1±5.0	79.7±3.1	-0.2	-16.1
Placebo then BS	87.8±3.9	85.4±3.6	85.1±3.4	90.2±3.7	-2.8	4.8
Treatment effect	-	-	-	-	-1.5	-5.6
95% confidence interval					-10.0 to 7.0	-14.4 to 4.2
Welch's t test					*p*=0.727	*p*=0.272
8-OHdG (ng/mL)						
BS then placebo	1.8±0.3	1.4±0.1	1.8±0.1	1.4±0.1	0	0
Placebo then BS	1.5±0.1	1.7±0.1	1.4±0.0	1.2±0.0	-0.1	-0.5
Treatment effect	-	-	-	-	4.6	-0.3
95% confidence interval					-0.4 to 0.2	-0.4 to -0.04
Welch's t test					*p*=0.354	*p*=0.011
Urine						
NO_2/NO_3 (μM)						
BS then placebo	28.2±10.1	16.8±5.6	22.2±6.2	28.9±10.8	-0.6	12.1
Placebo then BS	14.3±3.7	14.2±4.3	15.3±6.1	34.2±10.5	1.1	20
Treatment effect	-	-	-	-	-2.5	16
95% confidence interval					-16.3 to 12.0	-0.6 to 32.7
Welch's t test					*p*=0.762	*p*=0.029

HEL (nM)							
BS then placebo	108.0±34.2	83.2±20.0	136.1±35.7	103.4±15.4		28.1	20.2
Placebo then BS	112.7±48.7	161.7±79.9	120.1±28.6	124.7±26.4		7.4	-37.1
Treatment effect	-	-	-	-	-	17.8	-8.4
95% confidence interval						-58.5 to 94.6	-98.1 to 82.3
Welch's t test						*p*=0.636	*p*=0.858
MPO (ng/mL)							
BS then placebo	10.2±0.1	17.7±7.7	10.1±0.1	10.3±0.2		-0.1	-7.5
Placebo then BS	10.8±0.7	10.2±0.1	10.1±0.1	9.9±0.0		-0.7	-0.3
Treatment effect	-	-	-	-		-0.4	-3.9
95% confidence interval						-1.1 to 0.3	-11.9 to 4.1
Welch's t test						*p*=0.277	*p*=0.326
8-OHdG (ng/mL)							
BS then placebo	7.6±1.6	15.1±4.6	8.3±2.2	8.0±0.9		0.7	-7.0
Placebo then BS	11.1±2.9	9.9±2.2	7.0±2.1	10.9±1.1		-1.6	-2.9
Treatment effect	-	-	-	-	-	-0.5	-5.0
95% confidence interval						-5.2 to 4.1	-10.4 to 0.6
Welch's t test						*p*=0.403	*p*=0.039

BS; black soybean. Number of "BS then placebo" and "Placebo then BS" groups was 11 each and that of "Treatment effect" was 22.

Table S4. Supporting data for the effect of black soybean consumption on polyphenol concentration in the plasma (Figure 1).

**Test material**	**Placebo**		Black soybean			within-individual difference: BS-Placebo	
**Period**	**Start**	**End**	Start	End		Start	End
(−)-Epicatechin (nM)							
Aglycone							
BS then placebo	1.15±0.16	0.90±0.18	0.79±0.15	1.52±0.38		-0.36	0.62
Placebo then BS	2.60±0.51	2.64±0.57	2.51±0.43	3.63±0.59		-0.09	0.99
Treatment effect	-	-	-	-	-	-0.23	0.81
95% confidence interval						-1.4 to 0.8	0.2 to 1.8
Welch's t test						*p*=0.613	*p*=0.104
Conjugates							
BS then placebo	0.51±0.13	0.67±0.18	0.64±0.13	1.80±0.60		0.13	1.14
Placebo then BS	1.30±0.54	1.33±0.34	0.68±0.19	2.11±0.71		-0.62	0.78
Treatment effect	-	-	-	-	-	-0.24	0.96
95% confidence interval						-11.7 to 5.8	-0.5 to 10.2
Welch's t test						*p*=0.490	*p*=0.075
Daidzein (nM)							
Aglycone							
BS then placebo	0.008±0.002	0.007±0.002	0.005±0.002	0.015±0.004		-0.003	0.008
Placebo then BS	0.009±0.003	0.004±0.001	0.008±0.002	0.014±0.003		-0.001	0.01
Treatment effect	-	-	-	-		-0.002	0.009

95% confidence interval					-	-5.8 to 6.2	2.6 to 14.6
Welch's t test						*p*=0.9996	*p*=0.002
Conjugates							
BS then placebo	0.005 ±0.003	0.007 ±0.003	0.005 ±0.002	0.061 ±0.025		0	0.054
Placebo then BS	0.013 ±0.006	0.007 ±0.002	0.016 ±0.009	0.023 ±0.007		0.003	0.016
Treatment effect	-	-	-	-	-	0.002	0.035
95% confidence interval						-23.4 to 31.2	10.7 to 65.3
Welch's t test						*p*=0.999	*p*=0.003
Genistein (nM)							
Aglycone							
BS then placebo	0.002 ±0.001	0.002 ±0.001	0.005 ±0.002	0.005 ±0.001		0.003	0.003
Placebo then BS	0.005 ±0.004	0.006 ±0.005	0.008 ±0.006	0.010 ±0.006		0.003	0.004
Treatment effect	-	-	-	-	-	0.003	0.004
95% confidence interval						-15.7 to 20.2	-16.8 to 19.1
Welch's t test						*p*=0.988	*p*=0.998
Conjugates							
BS then placebo	0.088 ±0.051	0.101 ±0.048	0.039 ±0.021	0.225 ±0.061		-0.049	0.124
Placebo then BS	0.050 ±0.014	0.040 ±0.014	0.056 ±0.017	0.110 ±0.030		0.006	0.07
Treatment effect	-	-	-	-	-	-0.022	0.097
95% confidence interval						-59.4 to 125.6	5.4 to 190.4
Welch's t test						*p*=0.780	*p*=0.002
Glycitein (nM)							
Aglycone							
BS then placebo	0.005 ±0.001	0.008 ±0.002	0.005 ±0.002	0.007 ±0.002		0	-0.001
Placebo then BS	0.005 ±0.001	0.003 ±0.001	0.004 ±0.001	0.006 ±0.001		-0.001	0.003
Treatment effect	-	-	-	-	-	-0.001	0.001
95% confidence interval						-3.2 to 3.2	-1.8 to 4.7
Welch's t test						*p*=1.000	*p*=0.636
Conjugates							
BS then placebo	0.004 ±0.002	0.003 ±0.002	0.004 ±0.002	0.008 ±0.004		0	0.005
Placebo then BS	0.002 ±0.001	0.001 ±0.001	0.002 ±0.001	0.003 ±0.001		0	0.002
Treatment effect	-	-	-	-	-	0	0.004
95% confidence interval						-5.2 to 5.4	-1.1 to 9.5
Welch's t test						*p*=1.000	*p*=0.164
(*S*)-Equol (µM)							
Aglycone							
BS then placebo	0.002 ±0.002	0.001 ±0.001	0.010 ±0.004	0.013 ±0.004		0.008	0.012
Placebo then BS	0.006 ±0.002	0.003 ±0.001	0.003 ±0.001	0.008 ±0.002		-0.003	0.005
Treatment effect	-	-	-	-	-	0.003	0.009
95% confidence interval						-2.8 to 8.4	0.4 to 11.5
Welch's t test						*p*=0.549	*p*=0.031
Conjugates							
BS then placebo	0.016 ±0.015	0.018 ±0.013	0.008 ±0.006	0.058 ±0.047		-0.008	0.04
Placebo then BS	0.005 ±0.004	0.002 ±0.001	0.010 ±0.005	0.014 ±0.004		0.005	0.012
Treatment effect	-	-	-	-	-	-0.002	0.026

95% confidence interval	-38.3 to 43.0	-10.9 to 70.4
Welch's t test	*p*=0.999	*p*=0.225

BS; black soybean. Number of "BS then placebo" and "Placebo then BS" groups was 11 each and that of "Treatment effect" was 22.

Table S5A. Supporting data for the effect of black soybean consumption on polyphenol concentration in the urine (Figure 2).

**Test material**	**Placebo**		Black soybean			within-individual difference: BS-Placebo	
**Period**	**Start**	**End**	Start	End		Start	End
C3G (μM)							
Aglycone							
BS then placebo	1.781±0.231	2.083±0.336	1.421±0.204	2.927±1.224		-0.36	0.844
Placebo then BS	1.518±0.223	1.797±0.271	1.260±0.187	2.315±0.365		-0.258	0.518
Treatment effect	-	-	-	-	-	-0.309	0.681
95% confidence interval						-0.73 to 0.11	-0.68 to 2.04
Welch's t test						*p*=0.148	*p*=0.313
Conjugates							
BS then placebo	0.189±0.069	0.697±0.191	0.334±0.146	0.667±0.217		0.145	-0.03
Placebo then BS	0.323±0.060	0.430±0.227	0.278±0.053	0.384±0.125		-0.045	-0.046
Treatment effect	-	-	-	-	-	0.050	-0.038
95% confidence interval						-0.13 to 0.23	-0.43to 0.35
Welch's t test						*p*=0.579	*p*=0.846
(−)-Epicatechin (nM)							
Aglycone							
BS then placebo	0.349±0.117	0.383±0.073	0.292±0.063	1.316±0.754		-0.057	0.933
Placebo then BS	0.395±0.101	0.685±0.232	0.453±0.075	0.698±0.118		0.058	0.013
Treatment effect	-	-	-	-	-	0.001	0.473
95% confidence interval						-0.18 to 0.18	-0.35 to 1.29
Welch's t test						*p*=0.999	*p*=0.251
Conjugates							
BS then placebo	0.223±0.125	0.360±0.088	0.532±0.224	1.442±0.816		0.309	1.082
Placebo then BS	0.221±0.078	0.249±0.085	0.228±0.059	0.490±0.189		0.007	0.241
Treatment effect	-	-	-	-	-	0.158	0.662
95% confidence interval						-0.14 to 0.46	-0.22 to 1.54
Welch's t test						*p*=0.286	*p*=0.135
Procyanidin B2 (nM)							
Aglycone							
BS then placebo	0.101±0.054	0.076±0.018	0.032±0.005	0.130±0.041		-0.069	0.054
Placebo then BS	0.052±0.007	0.045±0.012	0.052±0.008	0.100±0.020		0	0.055
Treatment effect	-	-	-	-	-	-0.035	0.055
95% confidence interval						-0.09 to 0.02	0.003 to 0.105
Welch's t test						*p*=0.333	*p*=0.211
Conjugates							
BS then placebo	0.062±0.026	0.071±0.025	0.022±0.010	0.072±0.045		-0.04	0.001

Placebo then BS	0.011±0.002	0.020±0.005	0.021±0.006	0.099±0.035		0.01	0.079
Treatment effect	-	-	-	-	-	-0.015	0.04
95% confidence interval						-0.05 to 0.02	-0.02 to 0.10
Welch's t test						*p*=0.222	*p*=0.038
Procyanidin C1 (nM)							
Aglycone							
BS then placebo	0.009±0.003	0.008±0.002	0.009±0.002	0.024±0.012		0	0.016
Placebo then BS	0.010±0.003	0.014±0.004	0.009±0.003	0.016±0.003		-0.001	0.002
Treatment effect	-	-	-	-	-	-0.001	0.009
95% confidence interval						-0.005 to 0.005	-0.004 to 0.02
Welch's t test						*p*=0.972	*p*=0.180
Conjugates							
BS then placebo	0.002±0.001	0.003±0.001	0.008±0.007	0.015±0.010		0.006	0.012
Placebo then BS	0.003±0.001	0.003±0.001	0.003±0.002	0.004±0.001		0	0.001
Treatment effect	-	-	-	-	-	0.003	0.007
95% confidence interval						-0.005 to 0.010	-0.004 to 0.017
Welch's t test						*p*=0.479	*p*=0.208
Cinnamtannin A2 (nM)							
Aglycone							
BS then placebo	0.0005±0.0002	0.0003±0.0001	0.0014±0.0006	0.0030±0.0010		0.0009	0.0027
Placebo then BS	0.0007±0.0003	0.0016±0.0011	0.0007±0.0003	0.0006±0.0001		0	-0.001
Treatment effect	-	-	-	-	-	0.0005	0.0009
95% confidence interval						-0.001 to 0.001	-0.002 to 0.001
Welch's t test						*p*=0.630	*p*=0.282
Conjugates							
BS then placebo	0.0001±0.0001	0.0002±0.0001	0.0014±0.0011	0.0010±0.0006		0.0013	0.0008
Placebo then BS	0.0002±0.0001	0.0001±0.0001	0.004±0.0002	0.0002±0.0001		0.0002	0.0001
Treatment effect	-	-	-	-	-	0.0008	0.0004
95% confidence interval						0.0003 to 0.0016	0.0004 to 0.0029
Welch's t test						*p*=0.008	*p*=0.011

BS; black soybean. Number of "BS then placebo" and "Placebo then BS" groups was 11 each and that of "Treatment effect" was 22.

Table S5B. Supporting data for the effect of black soybean consumption on polyphenol concentration in the urine (Figure 2).

**Test material**	**Placebo**		Black soybean			within-individual difference: BS-Placebo	
**Period**	**Start**	**End**	Start	End		Start	End
Daidzin (nM)							
Aglycone							
BS then placebo	0.028±0.006	0.024±0.004	0.023±0.008	0.042±0.012		-0.005	0.018
Placebo then BS	0.025±0.004	0.021±0.006	0.012±0.003	0.025±0.005		-0.013	-0.004
Treatment effect	-	-	-	-	-	-0.009	0.007
95% confidence interval						-0.0199 to 0.0013	-0.0038 to 0.0243

Welch's t test						*p*=0.083	*p*=0.145
Conjugates							
BS then placebo	0.026±0.014	0.029±0.011	0.065±0.046	0.180±0.071		0.039	0.151
Placebo then BS	0.036±0.019	0.023±0.008	0.039±0.021	0.289±0.097		0.003	0.266
Treatment effect	-	-	-	-	-	0.021	0.209
95% confidence interval						-0.034 to 0.073	0.083 to 0.344
Welch's t test						*p*=0.466	*p*=0.999
Genistin (nM)							
Aglycone							
BS then placebo	0.005±0.003	0.002±0.002	0.002±0.001	0.017±0.007		-0.003	0.015
Placebo then BS	0.001±0.001	0.002±0.002	0.002±0.002	0.005±0.002		-0.001	0.003
Treatment effect	-	-	-	-	-	-0.002	0.009
95% confidence interval						-0.0046 to 0.0036	0.0005 to 0.0161
Welch's t test						*p*=0.811	*p*=0.037
Conjugates							
BS then placebo	0.037±0.031	0.041±0.040	0.003±0.002	0.203±0.105		-0.034	0.162
Placebo then BS	0.021±0.011	0.013±0.006	0.010±0.005	0.452±0.282		-0.011	0.439
Treatment effect	-	-	-	-	-	-0.023	0.301
95% confidence interval						-0.0533 to 0.0099	-0.0242 to 0.6520
Welch's t test						*p*=0.167	*p*=0.067
Glycitin (nM)							
Aglycone							
BS then placebo	0.074±0.018	0.087±0.019	0.108±0.025	0.152±0.056		0.034	0.065
Placebo then BS	0.070±0.017	0.076±0.013	0.040±0.010	0.150±0.031		-0.03	0.074
Treatment effect	-	-	-	-	-	0.002	0.07
95% confidence interval						-0.0394 to 0.0365	0.0055 to 0.1349
Welch's t test						*p*=0.941	*p*=0.035
Conjugates							
BS then placebo	0.058±0.021	0.024±0.014	0.120±0.076	0.141±0.056		0.062	0.117
Placebo then BS	0.059±0.025	0.067±0.027	0.056±0.025	0.418±0.194		-0.003	0.351
Treatment effect	-	-	-	-	-	-0.03	0.234
95% confidence interval						-0.0556 to 0.1075	0.0095 to 0.4815
Welch's t test						*p*=0.518	*p*=0.042
Daidzein (nM)							
Aglycone							
BS then placebo	3.124±1.171	1.288±5.538	2.435±0.588	10.426±3.921		-0.689	9.138
Placebo then BS	2.161±0.445	2.260±0.597	1.800±0.331	4.930±1.725		-0.361	2.67
Treatment effect	-	-	-	-	-	-0.002	0.009
95% confidence interval						-1.8449 to 0.8289	1.2599 to 9.9011
Welch's t test						*p*=0.443	*p*=0.014
Conjugates							
BS then placebo	23.286±9.862	12.622±0.003	9.555±7.466	26.985±6.492		-13.731	14.363
Placebo then BS	13.287±5.173	13.962±8.289	20.650±4.753	40.633±12.576		7.363	26.671
Treatment effect	-	-	-	-	-	-3.184	20.517
95% confidence interval						-15.782 to 11.523	2.7271 to 39.5389

Welch's t test						*p*=0.754	*p*=0.026
Genistein (nM)							
Aglycone							
BS then placebo	1.669±0.710	0.973±0.280	0.831±0.273	4.850±1.657		-0.838	3.877
Placebo then BS	0.974±0.372	0.549±0.136	1.080±0.289	2.586±1.532		0.106	2.037
Treatment effect	-	-	-	-	-	-0.366	2.957
95% confidence interval						-1.1889 to 0.5517	0.4948 to 5.2350
Welch's t test						*p*=0.460	*p*=0.020
Conjugates							
BS then placebo	15.772±7.714	11.925±5.228	11.504±8.302	34.483±10.833		-4.286	22.558
Placebo then BS	8.103±2.888	7.213±2.894	10.041±1.942	40.772±12.587		1.938	33.559
Treatment effect	-	-	-	-	-	-1.174	28.059
95% confidence interval						-11.687 to 9.978	10.528 to 46.689
Welch's t test						*p*=0.874	*p*=0.001
Glycitein (nM)							
Aglycone							
BS then placebo	1.327±0.497	1.166±0.302	1.021±0.365	2.959±0.666		-0.306	1.793
Placebo then BS	1.254±0.398	1.928±0.620	0.801±0.167	3.032±0.781		-0.453	1.104
Treatment effect	-	-	-	-	-	-0.38	1.449
95% confidence interval						-1.1127 to 0.3386	0.1347 to 2.6937
Welch's t test						*p*=0.285	*p*=0.031
Conjugates							
BS then placebo	4.554±1.809	6.379±2.974	3.554±2.728	20.283±5.996		-1.000	13.904
Placebo then BS	5.382±2.640	9.678±6.455	4.729±1.427	19.025±3.973		-0.653	9.347
Treatment effect	-	-	-	-	-	-0.827	11.626
95% confidence interval						-5.183 to 3.565	1.215 to 21.579
Welch's t test						*p*=0.710	*p*=0.029
(*S*)-Equol (μM)							
Aglycone							
BS then placebo	0.477±0.227	0.313±0.099	0.227±0.069	0.368±0.117		-0.25	0.055
Placebo then BS	0.277±0.062	0.299±0.075	0.212±0.054	0.524±0.157		-0.065	0.225
Treatment effect	-	-	-	-	-	-0.158	0.14
95% confidence interval						-0.3846 to 0.0878	-0.0887 to 0.3858
Welch's t test						*p*=0.207	*p*=0.211
Conjugates							
BS then placebo	6.561±5.442	6.553±5.222	2.393±1.893	9.898±7.537		-4.168	3.345
Placebo then BS	1.061±0.432	0.813±0.231	1.126±0.481	4.815±2.862		0.065	4.002
Treatment effect	-	-	-	-	-	-2.052	3.674
95% confidence interval						-7.2356 to 3.5553	-5.186 to 12.599
Welch's t test						*p*=0.488	*p*=0.402

BS; black soybean. Number of "BS then placebo" and "Placebo then BS" groups was 11 each and that of "Treatment effect" was 22.
